# Identification of a novel conserved HLA-A*0201-restricted epitope from the spike protein of SARS-CoV

**DOI:** 10.1186/1471-2172-10-61

**Published:** 2009-12-03

**Authors:** Yanbo Lv, Zhihua Ruan, Li Wang, Bing Ni, Yuzhang Wu

**Affiliations:** 1 Institute of Immunology, PLA, Third Military Medical University, 30 Gaotanyan Street, Chongqing 400038, PR China

## Abstract

**Background:**

The spike (S) protein is a major structural glycoprotein of coronavirus (CoV), the causal agent of severe acute respiratory syndrome (SARS). The S protein is a potent target for SARS-specific cell-mediated immune responses. However, the mechanism CoV pathogenesis in SARS and the role of special CTLs in virus clearance are still largely uncharacterized. Here, we describe a study that leads to the identification of a novel HLA-A*0201-restricted epitope from conserved regions of S protein.

**Results:**

First, different SARS-CoV sequences were analyzed to predict eight candidate peptides from conserved regions of the S protein based upon HLA-A*0201 binding and proteosomal cleavage. Four of eight candidate peptides were tested by HLA-A*0201 binding assays. Among the four candidate peptides, Sp8 (S_958-966_, VLNDILSRL) induced specific CTLs both *ex vivo *in PBLs of healthy HLA-A2^+ ^donors and in HLA-A2.1/K^b ^transgenic mice immunized with a plasmid encoding full-length S protein. The immunized mice released IFN-γ and lysed target cells upon stimulation with Sp8 peptide-pulsed autologous dendritic cells in comparison to other candidates.

**Conclusion:**

These results suggest that Sp8 is a naturally processed epitope. We propose that Sp8 epitope should help in the characterization of mechanisms of virus control and immunopathology in SARS-CoV infection.

## Background

Severe acute respiratory syndrome (SARS), a newly emerging infectious disease, is caused by a SARS-associated coronavirus (SARS-CoV) [1-3], which may originate from some wild animals [4]. After its first occurrence, SARS rapidly spread around the world along international air-travel routes, reaching all five continents and resulting in several hundreds of deaths [5]. The most recent epidemic of SARS occurred in Beijing and Anhui, China in April 2004 and originated from laboratory contamination (WHO update 7; see Further Information). Although the outbreaks seem to be over, SARS remains a safety concern because of the possible reintroduction of a SARS-like coronavirus (SL-CoV) into humans and the risk of an escape of SARS-CoV from laboratories [6-8]. More importantly, a new recombinant virus derived from human [9], swine and/or avian influenza virus, might re-emerge as a new SARS-CoV type, much like the recent emergence of a novel swine-origin influenza A (H1N1) in humans. Thus, it is essential to develop various and distinct strategies to combat this highly contagious disease.

The published literature reports that high titres of neutralizing antibodies and SARS-CoV-specific cytotoxic T lymphocyte (CTL) responses were detected in patients who had recovered from SARS [10,11], and the levels of those responses correlated well with disease outcome [12]. Hence, both humoral and cellular immune responses appear to be crucial for the clearance of SARS-CoV infection. Immune responses can be raised directly against several of the SARS-CoV proteins [13-15]. Targeting the spike (S) structural glycoprotein [12,16-18] in particular induces a robust immune response, suggesting it plays an important role in the systemic clearance of SARS-CoV [10]. The viral surface S protein is involved in host cell receptor recognition, virus attachment and entry [19]; adaptive evolution of S protein, thus, contributes to SARS-CoV overcoming the species barrier [20]. Hence, many vaccines and therapeutics against SARS-CoV target the S protein [19]. Considering that cytotoxic T-cell responses participate in the clearance of virus from recovered SARS patients and contribute to immunopathology in early stages of the disease [21], one of the most attractive S protein-based strategies proposes eliciting a SARS-CoV CTL response to clear the infection. To this end, a detailed understanding of the S protein-mediated CTL response is essential.

Development of effective treatments and vaccines against SARS-CoV depends upon the underlying mechanisms of various immune effectors in protective immunity and identification of the protective antigens recognized by each. Epitopes are the basic antigenic elements of virus structural proteins, which functionally induce the host cell-mediated immune response. Identification of the CTL-specific epitopes of SARS-CoV proteins could provide the basis for the development of SARS immunity-based treatments and aid in the understanding of mechanisms underlying SARS-CoV pathogenesis.

HLA-A*0201 is expressed 39-46% of all major ethnicities [22]. The identification of HLA-A*0201-restricted SARS-CoV/S CTL epitopes is an important contribution towards understanding the role of CTLs in SARS-CoV pathogenesis and protection. Currently, several CTL-specific SARS-CoV S protein epitopes have been identified in the context of HLA-A*0201, including S_411-420_, S_787-795_, S_978-986_, S_1042-1050_, S_1167-1175_, S_1203-1211 _and SSp-1 [23-26], and the H2 complex, including S_366-374_, S_436-443_, S_525-532 _and S_1031-1047 _[18,27]. It is likely that additional S protein CTL epitopes exist. S protein is relatively large in size and usage of different detection methods may result in the identification of novel S protein-derived epitopes. In turn, this data will provide advances towards understanding the mechanisms of SARS-CoV infection, and contribute to the development of future SARS-CoV infection intervention strategies.

In this study, we identified a novel SARS/S-specific, HLA-A*0201-restricted epitope that was conserved among SARS-CoV strains. Based on a binding affinity-based prediction and a proteosomal cleavage site prediction, we constructed a panel of potential HLA-A*0201-restricted CTL peptides from the S protein. Each candidate peptide was evaluated for its binding affinity to HLA-A*0201 molecules using the T2 cell-peptide binding test. We then evaluated the ability of HLA-A*0201 binding peptides to provoke CTL responses in peripheral blood lymphocytes (PBL). PBL preparations from major histocompatibility complex (MHC)-matched healthy donors or HLA-A2.1/K^b ^transgenic (Tg) mice, were incubated with dendritic cells (DCs) that had been pre-pulsed with the peptides of interest. We identified a novel SARS-CoV S protein-derived CTL epitope S_(958-966)_(VLNDILSRL) that was capable of priming the S protein-specific HLA-A2.1-restricted CTL response. The effective CTL response was evidenced by cell death of peptide-pulsed T2 and peptide-pulsed Jurkat-A2/K^b ^cells. The findings of this study should provide insight into the immunological characteristics of spike protein and provide an alternative strategy for the future development of SARS-CoV S protein CTL epitope-based vaccines.

## Results

### Selection of potential HLA-A*0201 binding peptides derived from SARS-CoV/S protein

**Figure 1:**
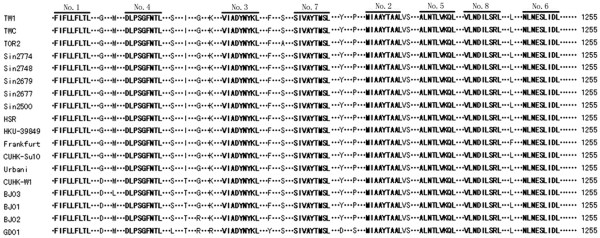
**Alignment of the putative amino acid sequences of S proteins from eighteen SARS-CoV strains**. SARS-CoV strains with GenBank accession numbers of nucleotide sequence in parentheses were as follows: BJ01 (AY278488), BJ02 (AY278487), BJ03 (AY278490), CUHK-Su10 (AY282752), CUHK-W1 (AY278554), Frankfurt 1 (AY291315), GD01 (AY278489), HKU-39849 (AY278491), HSR 1 (AY323977), Sin2500 (AY283794), Sin2677 (AY283795), Sin2679 (AY283796), Sin2748 (AY283797), Sin2774 (AY283798), TOR2 (AY274119), TW1 (AY291451), TWC (AY321118) and Urbani (AY278741). A dot among the individual sequences denoted nucleotides that are the same as the consensus. The candidate epitope peptides were shown in bold text.

We selected candidate CTL epitopes derived from SARS-CoV S protein by two criteria: (*i*) conservation between different strains of SARS-CoV to encompass as many SARS-CoV strains as possible, and (*ii*) high representation in the general population, i.e., HLA-A*0201-restricted. After alignment of the amino acid residues of S protein with eighteen SARS-CoV strains (Figure 1), the BJ01 strain S protein was selected to predict the S protein specific, HLA-A*0201-restricted CTL epitopes. Based on the presence of HLA-A*0201 binding motifs and the cleavage sites for proteasomes and immunoproteasomes, eight candidate peptides were predicted and synthesized (Figure 1 and Table 1), termed Sp1-8. These peptides were further verified as having an absence of shared sequence homology with the human or murine proteins using the BLAST search engine http://www.ncbi.nlm.nih.gov/blast/, to avoid any autoreactive response.

**Table 1:** HLA-A*0201 binding affinity of candidate epitope peptides on T2 cells.

Peptide	Denomination	Start position	Sequence	Score*	FI^†^
No.1	Sp1	2	FIFLLFLTL	24	0.5
No.2	Sp2	851	MIAAYTAAL	26	0.8
No.3	Sp3	404	VIADYNYKL	26	0.4
No.4	Sp4	208	DLPSGFNTL	24	0.1
No.5	Sp5	940	ALNTLVKQL	27	1.1
No.6	Sp6	1174	NLNESLIDL	27	1.1
No.7	Sp7	673	SIVAYTMSL	25	1.2
No.8	Sp8	958	VLNDILSRL	27	1.5

* HLA-A*0201-binding motif score from algorithm SYFPEITHI ≥ 24. The eight peptides were also predicted and selected using ProPred1, in which the threshold of HLA-A2-binding motif is 4% and the threshold of proteasomal and immunoproteasomal cleavage site is 8%.

† Increase of HLA-A*0201 molecules on T2 cell surface. FI = [(mean FITC fluorescence with the given peptide - mean FITC fluorescence without peptide)/(mean FITC fluorescence without peptide)]. FI > 1.0 indicates high-affinity peptides; FI ≤ 1.0, low-affinity peptides. HLA-A*0201-restricted peptide S_411-420 _was used as a positive control for HLA-A*0201-binding ability, while the H-2^b^-restricted peptide HBcAg_(131-140) _was used as a negative control.

To evaluate the binding affinity of these peptides to HLA-A*0201 molecules, a T2 cell-peptide binding test was used [28]. T2 cells lack the transporter associated with antigen processing (TAP), a key factor involved in endogenous antigen processing and presentation, causing the empty HLA-I molecules on the T2 cell surface to be very unstable and to degrade rapidly after cell surface presentation. However, when exogenous epitope peptides bind to the HLA-I molecules on the cell surface, they become stable [28,29]. Accordingly, the peptide-induced upregulation of HLA-I on TAP-deficient T2 cells is used to monitor peptide binding to class I molecules, which then indicates the binding affinity of peptides to HLA-I molecules. Higher-affinity peptides will induce more HLA-A*0201 expression on the cell surface than will lower-affinity peptides. As shown in Table 1, of the eight candidate peptides only Sp5, Sp6, Sp7 and Sp8 were high-affinity epitopes (FI = 1.1, 1.1, 1.2 and 1.5, respectively). The positive control peptide, S_411-420_, bound HLA-A*0201 strongly (FI = 1.5), whereas no binding was observed with the negative control HBcAg_(131-140) _peptide (FI = 0.1).

### *Ex vivo *generation of peptide-specific CD8^+ ^CTLs from healthy human donor PBLs

**Figure 2:**
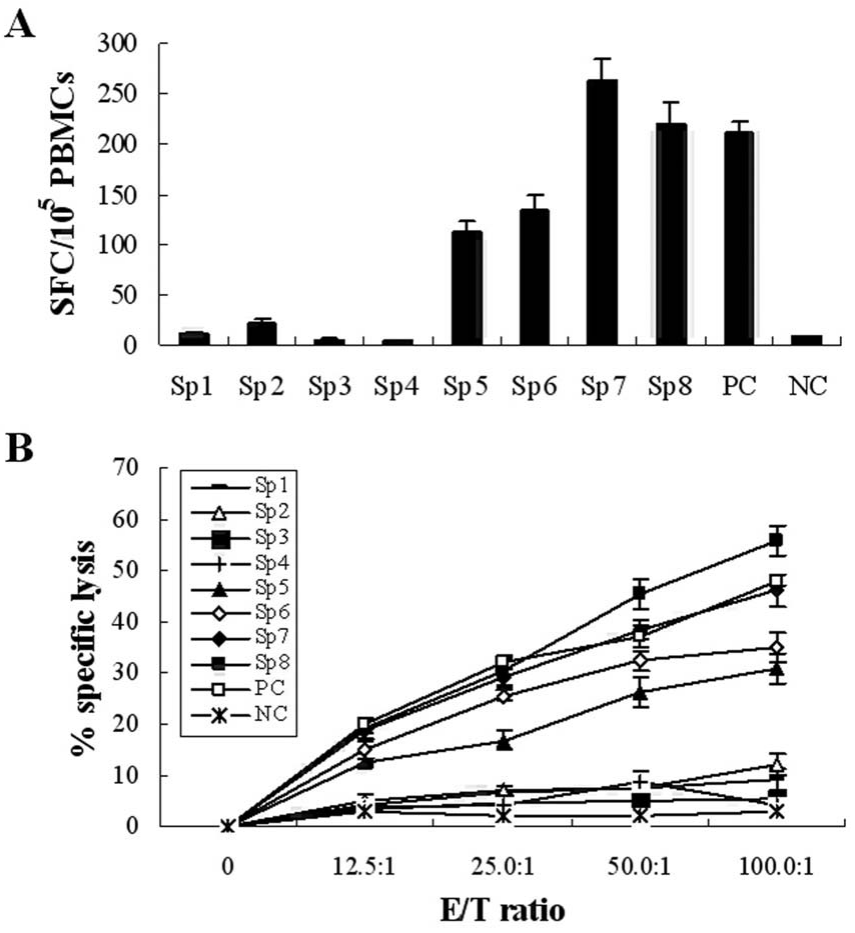
**Identification of candidate epitope peptides *ex vivo***. Panel A, Identification of candidate peptides with the PBLs of HLA-A2.1^+ ^healthy donors by ELISPOT assay. Autologous DCs were pulsed with 20 μg/ml of the indicated peptides and then used as stimulators for HLA-A2.1^+ ^PBLs in an IFN-γ release assay. Resulting CTLs were tested for IFN-γ release using an ELISPOT assay. Results presented are from three independent experiments and values are expressed as means ± SD. PC, positive control peptide S_411-420_; NC, negative control peptide HBcAg_(131-140)_. Panel B, Specific cytolysis of human CTLs induced by peptides-loaded DCs *ex vivo*. Peptide-specific CTLs were generated from the PBLs of HLA-A2.1^+ ^healthy donors through two rounds of stimulation with eight different peptide-pulsed DCs, respectively. Resulting CTLs were tested for peptide-specific lysis using a standard 4-hour ^51^Cr release assay. Results presented are from three independent experiments. Data is expressed as means ± SD. E/T ratio, effector cell to target cell ratio.

To investigate the capacity of candidate peptides to mobilize a human CTL repertoire, HLA-A2^+ ^PBLs from ten HLA-A2^+ ^donors were stimulated *in vitro *by DCs loaded with the eight candidate peptides, positive control peptides S_411-420_, or negative control peptides HBcAg_(131-140)_. T2 cells loaded with each of peptides were used as target cells in cytotoxicity assays. Of the eight peptides tested, Sp6, Sp7 and Sp8 induced more CD8^+ ^T-cells that specifically produced IFN-γ in response to DCs pulsed with the relevant peptides or positive control peptide in comparison to other groups (Figure 2A). Furthermore, these CD8^+ ^T cells could lyse the T2 cells loaded with relevant peptides or positive control peptide more efficiently than other groups (Figure 2B). The cytolysis observed were specific because the CTLs could not lyse T2 cells loaded with irrelevant peptides (Figure 2B). Due to the limited induction of IFN-γ secreting T cell frequency and the low CTL cytolysis ability, groups other than Sp6, Sp7 and Sp8 were excluded from further study. The *ex vivo *results showed the existence of functional anti-SARS-CoV/S CTL precursors in the peripheral T cell repertoire of healthy donors. Furthermore, SARS-CoV/S-derived peptides Sp6, Sp7 and Sp8 could not only induce the increased S protein specific IFN-γ secreting T cell frequency but also the enhanced cytolytic capacity of these CTLs.

### *In vivo *induction of peptide-specific CD8^+ ^CTLs in HLA-A2.1/K^b ^transgenic mice

**Figure 3:**
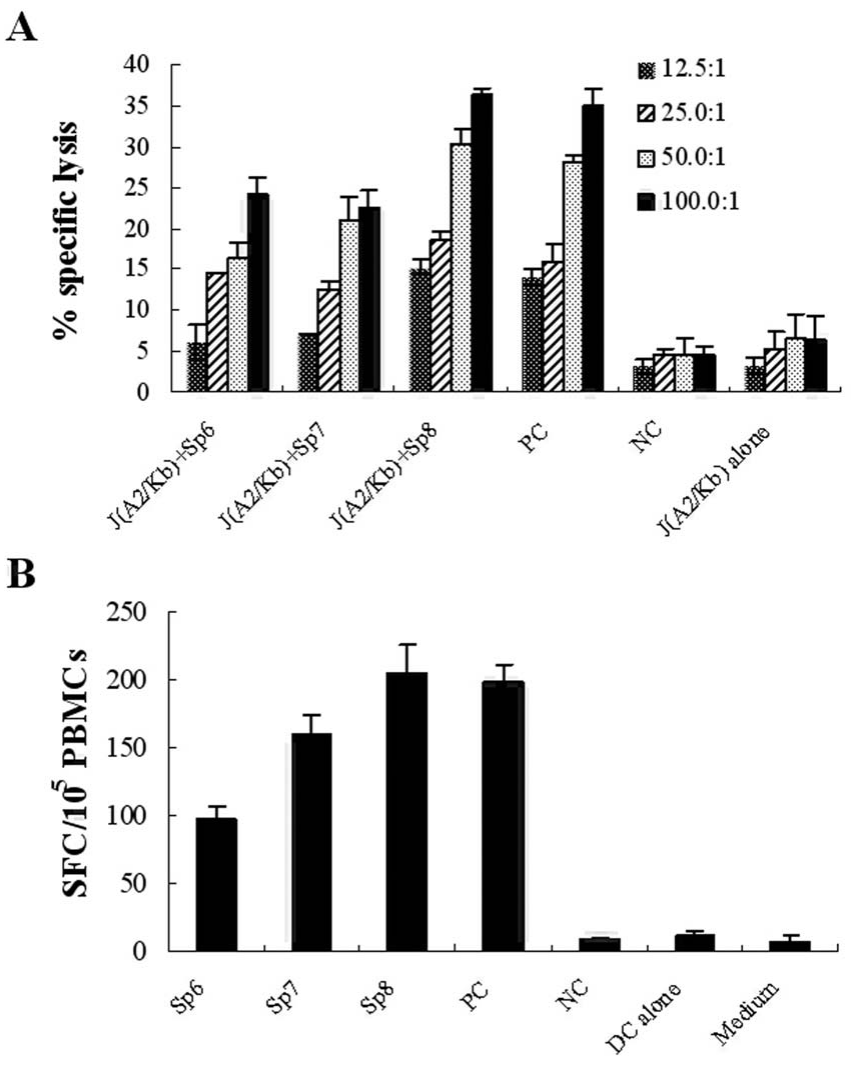
**SARS-CoV S protein specific CTLs in DNA vaccine-immunized HLA-A2.1/K^b^-Tg mice**. Panel A, Specific cytolysis of CTLs induced by the candidate peptides in HLA-A2.1/K^b ^transgenic mice. Splenic single-cell suspensions from S/pVAX1-immunized mice were harvested and re-stimulated with peptide-pulsed mouse bone marrow-derived DCs *in vitro *to act as effector cells. The J(A2/kb) cells pulsed with peptides were used as targets. Cytotoxic activity was determined in a standard ^51^Cr release assay at the indicated E/T ratios. Panel B, Frequency of IFN-γ producing cells induced by the indicated SARS S-derived candidate peptides in HLA-A2.1/K^b ^transgenic mice. Bulk CTLs from immunized mice were co-cultured with autologous DCs in the presence of each peptide at a final concentration of 20 μg/ml. The secretion of peptide-specific IFN-γ was analyzed using ELISPOT assays. Results presented were from three independent experiments. Data is expressed as means ± SD. E/T ratio, effector cell to target cell ratio.

To further address whether the immunogenic candidate peptide is naturally processed and presented, HLA-A2.1/K^b ^transgenic mice were immunized with S/pVAX1 plasmid containing a full-length cDNA encoding the SARS-CoV/S protein. Splenocytes were collected from mice seven days after four weekly injections with S/pVAX1, and re-stimulated *ex vivo *by mouse bone marrow-derived DCs loaded with the candidate peptides, the positive peptide S_411-420_, the irrelevant peptides HBcAg_(131-140)_, or DCs alone, for an additional 6 days. Investigation of IFN-γ production and the cytolytic ability of the effector CTL cells were carried out following the re-stimulation. The J(A2/k^b^) cells loaded with the corresponding peptides were used as targets in cytotoxicity assays. As shown in Figure 3A, CTLs from S/pVAX1-immunized mice were able to lyse three candidate peptides-pulsed J(A2/k^b^) cells but did not lyse J(A2/k^b^) cells alone or J(A2/k^b^) cells loaded with irrelevant peptide HBcAg_(131-140) _at any E/T ratio. Of the three candidate peptides tested, Sp8 exhibited the most lytic capacity at each E/T ratio, which was comparable to the positive control peptide (Figure 3A).

In accordance with results from the cytolytic assays, bulk CTLs released IFN-γ only in response to DCs pulsed with Sp6, Sp7, Sp8 and the positive control peptide, but not to those pulsed with irrelevant peptide HBcAg_(131-140) _or DCs alone (Figure 3B). Again, among the three tested candidate peptides, Sp8 released the most IFN-γ following peptide stimulation (Figure 3B).

## Discussion

It is known that SARS-CoV can induce a strong specific CTL response in infected patients, besides high titres of neutralizing antibodies [10,11]. Furthermore, the CTL response levels correlate with disease outcome [12], suggesting CTL response is crucial for the clearance of SARS-CoV. Among all the encoded proteins in the SARS-CoV genome, S protein is currently considered the most important target to prime the host immune response [12,16-18]. It has been reported that an inflammatory cell influx of airway macrophages and a massive release of cytokines occur during the peak of SARS infection [30]. Thus, it is reasonable to investigate the underlying mechanism of specific CTL response induced by SARS-CoV S protein during SARS-CoV infection which may have a positive role in SARS-CoV clearance or a negative role in SARS-CoV immunopathogenesis.

In this study, we predicted and validated a novel CTL epitope of SARS-CoV S protein. We used two prediction systems to identify candidate CTL epitopes of S protein (i.e., HLA-A2-binding peptide prediction method combined with a proteosomal cleavage site prediction system) to improve prediction accuracy. The eight predicted peptides were then verified via MHC peptide binding assay (Table 1). Among the eight candidate peptides, Sp5, Sp6, Sp7 and Sp8 exhibited the highest capacity to induce more potent CTLs secreting IFN-γ and to lyse target cells from HLA-A*0201-matched healthy donor PBLs (Figure 2). Further *in vivo *investigation showed that plasmid encoding the full-length SARS-CoV S gene elicited strong CTL response in HLA-A2.1/K^b ^transgenic mice. These CTLs could produce substantial amounts of IFN-γ and kill target cells in a peptide-specific and HLA-A*0201-restricted manner (Figure 3), suggesting the predicted candidate peptides were native epitopes, capable of priming CTL responses *in vivo*. We found that candidate peptide Sp8 held the greatest ability to secrete IFN-γ and kill target cells *in vivo *(Figure 3). Another candidate peptide, Sp7, failed to induce the most potent peptide specific CTLs in Tg mice (Figure 3), despite it having had the highest such ability in comparison to the rest of the *in vitro *stimulation set (Figure 2), indicating the need for candidate peptides to be biofunctionally validated *in vivo*.

To date, several CTL epitopes of SARS-CoV S protein have been identified in the context of HLA-A*0201, including S_411-420_, S_787-795_, S_978-986_, S_1042-1050_, S_1167-1175_, S_1203-1211 _and SSp-1 [23-26], or of the H2 complex, including S_366-374_, S_436-443_, S_525-532 _and S_1031-1047 _[18,27]. In this study, we predicted and validated a novel CTL epitope of SARS-CoV S protein, Sp8 (S_958_, VLNDILSRL). This may be due to the unique predictive methods used in our study. We combined strategies for prediction (i.e., HLA-A2-binding peptide prediction method combined with a proteosomal cleavage site prediction system). Previous studies used single methods, such as HLA peptide binding prediction or overlapping peptide strategy [18,23-27], suggesting different prediction strategies might lead to different results. In any case, the predicted candidate peptides require additional validation methods to ensure accuracy.

In our study, we also determined that among the eight peptides we predicted, four could potent prime CTLs to produce significant IFN-γ and lyse target cells; although, Sp8 peptide exhibited the most potency for CTL priming. However, Zhou *et al*. reported that they only found one predicted peptide that could stimulate IFN-γ secretion and target cell lysis [26]. This may reflect the different stimulators used in these studies; Zhou used peptides to stimulate the effector cells directly while we used DCs loaded with the candidate peptides.

We argue for the use of DCs as stimulator cells in *ex vivo *study because DCs are the most potent APCs for priming T cells, and they not only present peptides to T cells but also provide many important co-stimulatory signals. Moreover, under *in vivo *conditions DCs present peptides to T cells in the context of MHC molecules. Thus, using DCs as *ex vivo *stimulator cells most closely mimics the *in vivo *context.

## Conclusion

Our study has identified a novel conserved HLA-A*0201-restricted epitope from the spike protein of SARS-CoV. We propose that the newly identified epitope could be used for evaluation of SARS-CoV-specific CD8^+ ^T-cell responses during the course of SARS infection and treatment. This epitope should also aid in the characterization of virus control mechanisms and immunopathology of SARS-CoV infection. Ultimately, our findings may be relevant to the development of ethnically unbiased, widely applicable immunotherapeutic approaches for SARS disease.

## Methods

### Sequence alignments

Nucleotide sequences of SARS-CoV strains were analyzed using BioEdit version 5.0.9 software suite. The corresponding amino acid sequences of S protein were then aligned with Clustal W http://www.ebi.ac.uk/clustalw/. SARS-CoV strains used were as follows, with the nucleotide sequence GenBank accession numbers in parentheses: BJ01 (AY278488), BJ02 (AY278487), BJ03 (AY278490), CUHK-Su10 (AY282752), CUHK-W1 (AY278554), Frankfurt 1 (AY291315), GD01 (AY278489), HKU-39849 (AY278491), HSR 1 (AY323977), Sin2500 (AY283794), Sin2677 (AY283795), Sin2679 (AY283796), Sin2748 (AY283797), Sin2774 (AY283798), TOR2 (AY274119), TW1 (AY291451), TWC (AY321118) and Urbani (AY278741).

### Peptides

To identify potential HLA-A*0201-binding peptides within the S protein of the SARS-CoV (BJ01) strain, a combination of two computer algorithms was utilized. The predictive algorithm, "ProPred1"[31], is a matrix-based method that allows the prediction of MHC binding sites in an antigenic sequence for 47 MHC class-I alleles. We restricted our analysis to the HLA-A2 allele, since it is prevalent in a large percentage of all major ethnicities and it is the most extensively studied HLA class-I antigen [22]. ProPred1 also allows the prediction of the standard proteasomal and immunoproteasomales cleavage sites in an antigenic sequence. The simultaneous prediction of MHC binding and proteasomal cleavage sites in an antigenic sequence leads to the identification of potential T-cell epitopes. The second algorithm, "SYFPEITHI", was developed by H. G. Rammensee *et al *[32], and ranks peptides according to a score that takes into account the presence of primary and secondary MHC-binding anchor residues. The 9 mer peptides with a score exceeding 24 were selected in "SYFPEITHI".

The amino acid sequence of SARS-CoV/S (BJ01) was analyzed on both of the computer programs for the existence of 9-amino acid peptides predicted to bind to HLA-A2. The candidates peptides were synthesized at SHENYOU Biotech (Shanghai, China) and purified by reverse phase HPLC to > 95%, as confirmed by mass spectrometry. The published HLA-A*0201-restricted peptide S_411-420 _(KLPDDFMGCV) derived from the S protein of SARS-CoV [26] was used as a positive control for HLA-A*0201-binding ability, and the HBcAg-derived H-2^b^-restricted peptide HBcAg_(131-140) _(AYRPPNAPIL) was used as a negative control. Lyophilized peptides were dissolved in PBS at a concentration of 1 mg/ml and stored in aliquots at -20°C.

### Cells and Cell Culture

HLA-A2^+ ^individuals were selected by flow cytometry screening using the anti-HLA-A2 monoclonal antibody BB7.2. Buffy coats from HLA-A2^+ ^normal donors were purchased from Southwest Hospital (Third Military Medical University, Chongqing, China). PBL from an HLA-A2^+ ^healthy donor were separated on Ficoll-Hypaque density gradients (TBD, Inc, Tianjin, China), washed three times in phosphate-buffered saline (PBS), resuspended in RPMI1640 medium (Gibco, BRL) supplemented with L-glutamine (10 mg/ml), penicillin (5 × 10^4^U/L), streptomycin (50 mg/L) and 10% fetal calf serum (FCS), and plated in 6-well plates at 4 × 10^6 ^cells per well.

Human TAP-deficient T2 cell line and BB7.2 cell line producing mAb against HLA-A2 were purchased from American Type Culture Collection. T2 cell line was maintained in RPMI1640 medium supplemented with 20% fetal bovine serum and 100 μg/ml penicillin/streptomycin. BB7.2 cell line was maintained in DMEM containing 10% FCS, 4 μg/L glucose, penicillin (5 × 10^4^U/L) and streptomycin (50 mg/L). Jurkat-A2/K^b ^cells, a generous gift from Dr. W. Martin Kast (the Norris Comprehensive Cancer Center, Los Angeles, CA) and Dr. Jehad Charo (the Max Delbruck Center for Medicine, Berlin, Germany), were transfected with the HLA chimeric molecule containing the α1 and α2 domains from human HLA-A*0201 and α3 from mouse H-2K^b^, to serve as a model system of HLA restricted responses [33]. The Jurkat-A2/K^b ^(J(A2/kb)) cell line was maintained in RPMI1640 medium (Gibco, BRL) plus 10% calf serum and supplemented with 4 μg/L glucose, penicillin (5 × 10^4 ^U/L) and streptomycin (50 mg/L). All cell lines mentioned above were kept at 37°C in a humidified atmosphere of 5% CO_2 _in air.

### Animals

HLA-A2.1/K^b ^transgenic (Tg) mice were purchased from the Jackson Laboratory (Bar harbor, ME). For experimental purposes, six to eight week-old mice were used. Cell surface HLA-A*0201 expression was assessed by flow cytometry using fluorescein isothiocyanate (FITC)-labeled HLA-A2-specific mAb BB7.2 (Sterotec Ltd, Oxford, UK). Mice were kept in SPF animal care facilities and all experiments were performed according to the guidelines in the Institutional Animal Committee of TMMU.

### Binding assay of candidate peptides to HLA-A2

All candidate peptides were tested individually for their capacity to bind to HLA-A2 molecules on the surface of human TAP-deficient T2 cells [28]. Briefly, T2 cells were incubated with 20 μg/ml candidate peptides and 3 μg/ml human β2-microglobulin (Sigma, St Louis, MO) in serum-free RPMI1640 for 18 hours at 37°C in a 5% CO_2 _atmosphere. Expression of HLA-A*0201 on T2 cells was then determined by staining with FITC-conjugated anti-HLA-A2 mAb BB7.2 and data analyzed using a FACSCalibur flow cytometer (Becton Dickinson, Mountain View, CA) and CellQuest software (Becton Dickinson). The published peptide S_411-420 _and HBcAg_(131-140) _served as positive and negative control, respectively. The former is known to bind to HLA-A2 molecule with high affinity, the latter has been identified as mouse H2K^d ^epitope that has little binding affinity with HLA-A2 molecule. The fluorescence index (FI) was calculated as follows: FI = [(mean FITC fluorescence with the given peptide - mean FITC fluorescence without peptide)/(mean FITC fluorescence without peptide)]. Peptides with an FI more than 1 were regarded as high-affinity epitopes.

### Generation of CTLs in healthy donors

Human peripheral blood monocyte-derived DCs were generated as described previously [28] with minor modifications. Briefly, human PBLs were suspended in serum-free RPMI1640 and allowed to adhere to 6-well plates at a final concentration of 1 × 10^7 ^cells/3 ml/well and cultured in 5% CO2 at 37°C. After 2 hours, non-adherent cells were gently removed with warm medium. The resulting adherent cells were cultured in RPMI1640 medium supplemented with 10% FCS, 20 ng/ml recombinant human interleukin-4 (IL-4) (R&D Systems, Minneapolis, MN) and 800 U/ml recombinant human granulocyte-macrophage colony stimulating factor (GM-CSF; Sandoz, Basel, Switzerland) in 5% CO_2 _at 37°C. Every two days, one-half of the medium was replaced by fresh medium containing double concentration of GM-CSF and IL-4 as indicated above. Cell suspensions were collected for analysis of surface phenotype at different stages of development. After five days of culture, DCs were harvested for subsequent experiments (90% pure as confirmed by analysis of relatively DC-specific phenotype and with a typical DC morphology). 10 ng/ml recombinant human tumor necrosis factor (TNF-α, Peprotech, Rocky Hill, NJ) was added to the medium to induce phenotypic and functional maturation. Then, 48 hours later, DCs were used to prime autologous PBLs as follows, DCs were pulsed with 20 μg/ml peptide in the presence of 3 μg/ml β2-microglobulin at 37°C for 5 hours and irradiated at 30 Gy before use. PBLs (2 × 10^6 ^cells/3 ml culture medium) were co-cultured with 2 × 10^5 ^peptide-pulsed irradiated autologous DCs in a 6-well plate in the presence of 10 ng/ml recombinant human interleukin-7 (IL-7; Peprotech). After 24 to 48 hours 20 IU/ml human interleukin-2 (IL-2, Sigma) was added to the culture medium. Lymphocytes were re-stimulated each week in the same manner. Three days after the second round of re-stimulation, induced cells were harvested and tested by cytokine determination and cytotoxicity assays.

### Generation of CTLs in HLA-A2.1/K^b ^transgenic mice

A plasmid S/pVAX1 encoding SARS-CoV S protein was constructed and used to immunize the HLA-A2.1/K^b ^transgenic mice at a dose of 100 μg (in 100 μl of PBS) of plasmid S/pVAX1 by injection into tibialis anterior muscles. Mice were re-inoculated four times every seven days under the same conditions. In this study, bone marrow-derived DCs were generated from transgenic mice as previously described [34,35] with some modification. DCs were pulsed with 20 μg/ml peptide in the presence of 3 μg/ml β2-microglobulin at 37°C for 5 hours and irradiated at 30 Gy before use. Spleens were aseptically removed after the final scheduled immunization. Splenic single-cell suspensions were then harvested and cultured in 6-well plates at a density of 1 × 10^7 ^cells/3 ml/well, in the presence of 1 × 10^5 ^peptide-loaded irradiated syngeneic DCs. On day six of culture, induced cells were harvested and tested by cytokine determination and cytotoxicity assays.

### Cytotoxicity assays

Cytotoxic activity of CTLs was determined in a standard 4-hour ^51^Cr release assay as previously described [36] with some modification. In human cytotoxicity assays, DCs derived from a healthy HLA-A2^+ ^donor were incubated with each of candidate peptides and used to stimulate autologous healthy HLA-A2^+ ^donor PBLs. T2 cells loaded with the relevant peptides were used as target cells in cytotoxicity assays. As a positive control group, HLA-A2^+ ^PBLs were stimulated with S_411-420_-pulsed autologous DCs. HLA-A2^+ ^PBLs stimulated with HBcAg_(131-140)_-pulsed autologous DCs served as the negative control group. In DNA-immunized mice cytotoxicity assays, the target cells were the J(A2/kb) cells loaded with the candidate peptides, the positive control peptide S_411-420_, the irrelevant peptides HBcAg_(131-140)_, and J(A2/kb) cells alone.

First, T2 cells and/or J(A2/kb) cells were loaded with 20 μg/ml peptides and 3 μg/ml human β2-microglobulins and incubated at 37°C for 2 hours. Then peptide-pulsed T2 cells and/or J(A2/kb) cells were labeled with ^51^Cr sodium chromate (Na^51^CrO_4_, Perkin-Elmer Life Science, Boston, MA) for 90 minutes at 37°C. ^51^Cr-labeled target cells were washed three times and mixed with graded doses of effectors in 96-well plates. After incubation at 37°C for 4 hours, a total of 100 μl supernatant was collected from each well and radioactivity was counted with a gamma counter. Each assay was performed in triplicate. Percent specific lysis was determined according to the following formula: percent specific lysis = [(mean experimental cpm - mean spontaneous cpm)/(mean maximum cpm - mean spontaneous cpm)] × 100%. Spontaneous and maximum releases were determined by incubating the labeled targets with medium alone or 1 M HCl, respectively. Spontaneous release was always < 15% of maximum release.

### Enzyme-linked immunosorbent spot (ELISPOT) assay

ELISPOT assay was performed using a commercially available kit (U-CyTech, Netherlands) according to the manufacturer's instructions and published literature [37] with some modification. Autologous DCs were pulsed with 20 μg/ml candidate peptides and used as stimulators for HLA-A2.1^+ ^PBLs from the immunized mice. Effector cells (1 × 10^5^) and stimulator cells (1 × 10^5^) were seeded into 96-well polyvinylidene fluoride (PVDF)-backed microplates pre-coated with anti-IFN-γ mAb. After incubation at 37°C for 48 hours, cells were removed and plates processed as described in the instruction. Resulting spots were counted with a stereomicroscope (Carl Zeiss, Thornwood, NY) under magnifications of ×20 to ×40. Only brown and/or blue colored spots with fuzzy borders were scored as spot-forming cells (SFCs). As a positive control, S_411-420_-loaded DCs were used as stimulator cells. The HBcAg_(131-140)_-loaded DCs, DCs alone, and medium were used as negative controls. Negative control values were always < 20 SFC per 10^6 ^input cells. Results were considered positive when at least 120 SFC/10^6 ^PBL were detected. Each assay was run in triplicate and results were representative of three experiments.

## Authors' contributions

YL carried out the cell and animal manipulation, the CTL cytolysis assay. ZR carried out the immunoassays. LW participated in the design of the study and performed the statistical analysis. BN and YW conceived of the study, and participated in its design and coordination and helped to draft the manuscript. All authors read and approved the final manuscript.
